# A Cross-Reactive Monoclonal Antibody Against Neuraminidases of Both H9N2 and H3N2 Influenza Viruses Shows Protection in Mice Challenging Models

**DOI:** 10.3389/fmicb.2021.730449

**Published:** 2021-09-27

**Authors:** Fei Wang, Zhimin Wan, Jinsen Wu, Yajuan Wang, Hui Fu, Hongxia Shao, Kun Qian, Wei Gao, Jianqiang Ye, Aijian Qin

**Affiliations:** ^1^Ministry of Education Key Lab for Avian Preventive Medicine, Yangzhou University, Yangzhou, China; ^2^Jiangsu Co-innovation Center for Prevention and Control of Important Animal Infectious Diseases and Zoonoses, Yangzhou, China; ^3^Joint International Research Laboratory of Agriculture and Agri-Product Safety of Ministry of Education of China, Yangzhou University, Yangzhou, China

**Keywords:** monoclonal antibody, neuraminidase, H9N2, H3N2, influenza virus, cross-react, protection

## Abstract

Neuraminidases (NAs) of H9N2 avian influenza virus (AIV) and H3N2 human seasonal influenza virus (HSIV) share similar antigenic structures. However, there are few reports on epitopes shared by these two NAs. We previously reported a monoclonal antibody (mAb) 1G8 against the NA of H9N2 AIV with neuraminidase inhibition (NI) ability. In this study, 1G8 was shown to cross-react with and inhibit the NA of H3N2 HSIV. In a passive transfer experiment, 1G8 provided protection to mice challenged with rescued H1N2 viruses carrying H9N2 NA or H3N2 NA. Mutation at amino acid position 199 was also selected and proved to be crucial for H3N2 HSIV to escape from mAb 1G8. Moreover, we found that residue 199 contributed to inducing broad protective antibodies without the influence of the *N*-linked glycosylation at amino acid position 200 in NAs. Residues as residue 199, which are not shielded by glycosylation modification, would form ideal epitopes for developing universal vaccine and protective antibodies.

## Introduction

H9N2 subtype avian influenza virus (AIV) is one of the most prevalent influenza viruses in poultry and has caused substantial economic loss in poultry production (Bi et al., 2020; Carnaccini and Perez, 2020). Although H9N2 AIV infections in humans were rarely reported, the neuraminidase (NA) and other internal genes have been involved in generation of many highly pathogenic influenza viruses (Lin et al., 2000; Gao et al., 2013; Gu et al., 2014; Jianzhong et al., 2014; Liu et al., 2018). H3N2 subtype influenza virus is a human seasonal influenza virus (HSIV) and predominates in recent influenza seasons. H3N2 HSIV infection can cause severe respiratory system injury in humans, especially in the elderly (Kissling et al., 2017). Although H9N2 AIV and H3N2 HSIV share the same N2 subtype, there were few reports on same antigenic epitopes between them but universal epitopes among all subtypes of NAs (Doyle et al., 2013; Stadlbauer et al., 2019).

Neuraminidase is an important surface protein and antigen of influenza virus, which is a tetramer structure and plays an important role in helping newborn virus release from infected cells (Varghese et al., 1983). In the case of preexisted vaccination, antibodies targeting the NA active center can block the releasing of newborn virus (Eichelberger and Wan, 2015). However, as flexible as hemagglutinin (HA), NA can also escape from antibodies and inhibitors by amino acid mutations (Sandbulte et al., 2011; Wang et al., 2021). Many amino acid positions of H9N2 NA have been reported to be involved in escape from monoclonal antibodies (mAbs) and antibody pressure induced by vaccines (Wang et al., 2021). To control H3N2 HSIV, the best-matched strains for vaccination are updated every year, but antigenic drifts in NA have also been reported to help virus evade from the humoral immunity (Wan et al., 2019; Powell and Pekosz, 2020).

Conserved epitopes in HA and NA are crucial for developing universal influenza vaccines (Krammer and Palese, 2013; Wohlbold and Krammer, 2014). However, even if HA or NA belongs to the same subtype, the evolutionary directions might be different because of being prevalent in diverse hosts and exposed to various vaccinations (Lee et al., 2019). H9N2 AIV and H3N2 HSIV share the same N2 subtype and homologous sequences, while there were few reports on similar antigenic structure between them. In the present study, mAb 1G8 against the NA of H9N2 AIV was proved to cross-react with the NA of H3N2 HSIV and protect mice from viral challenge. Our research may facilitate the development of protective antibodies against N2 subtype AIV and HSIV in future.

## Materials and Methods

### Viruses, Cells, and Monoclonal Antibodies

A/Chicken/Jiangsu/H1/2019 (JSH1) H9N2 virus (accession numbers: MN515351 and MT459203) was isolated from poultry in China (Wang et al., 2021). A/Canine/Jiangsu/06/2010 (JS06) H3N2 virus (accession number: JN247616 to JN247623) was isolated from canine in China (Lin et al., 2012). All viruses were grown in 9-day-old embryonated SPF chicken eggs. Allantoic fluid of each virus was harvested at 120 h postinoculation and stored at −70°C. Madin-Darby canine kidney (MDCK) cells, African green monkey kidney-originated COS-1 cells, and human lung carcinomatous A549 cells were maintained in DMEM supplemented with 10% FBS at 37°C in 5% CO_2_. mAbs 1G8 and A7E6 were prepared as previously described (Wang et al., 2021). Ascitic fluid of each mAb was prepared in 8-week-old mice and purified with protein G column (GE, Boston, MA, United States).

### Reassortant Viruses Rescued by Reverse Genetics

Reassortant viruses were rescued by reverse genetics as previously reported (Shao et al., 2015). NA gene of A/Chicken/Jiangsu/H1/2019 (JSH1) H9N2 virus was amplified by reverse transcription-polymerase chain reaction (RT-PCR). NA gene of A/Beijing/PUMCH06/2017 (PUMCH06) H3N2 virus (accession number: MG759370.1) was synthetized by GenScript Co., Ltd., Nanjing, China. The NA genes and the other seven genes (HA, PA, PB1, PB2, NP, NS, and M) of A/Puerto Rico/8/34 (PR8) H1N1 virus were cloned into the pDP2002 vector by the Exnase II (Vazyme, Nanjing, China). The reassortant viruses rgH1N2(JSH1) and rgH1N2(PUMCH06) were rescued by transfection in COS-1 cells. Briefly, 1 μg of each plasmid was transfected into COS-1 cells with 16 μl of TransIT^®^-LT1 Transfection Reagent (Mirus, Madison, WI, United States). The culture medium was changed into Opti-MEM with 2 μg/ml TPCK-Trypsin at 6 h posttransfection. The recued viruses in supernatants of transfected cells were collected at 72 h posttransfection. Nine-day-old SPF eggs were used for expended culture of rescued viruses. Allantoic fluid of each virus was collected on the fifth day postinoculation and preserved at −70°C for further research.

### Immunofluorescence Assay

Briefly, COS-1 cells infected with viruses were fixed with cold acetone-alcohol at 48 h post-infection. After incubation of mAb 1G8 for 30 min, the fixed cells were washed three times with PBS and incubated with FITC conjugated goat anti-mouse-IgG(H + L) antibody (Jackson Immunoresearch, PA, United States) as secondary antibody. After 30 min, the cells were washed three times again and observed under inverted fluorescence microscopy (Olympus, Tokyo, Japan).

### Viral Growth Kinetics

A549 cells in 6-well plates were infected with rgH1N2(JSH1) and rgH1N2(PUMCH06) at a multiplicity of infection (MOI) of 0.01, respectively. The supernatants from the infected cells were collected at 12, 24, 36, 48, 60, and 72 h post-infection, and the viruses were titrated by median tissue infective dose (TCID_5__0_) assay in MDCK cells as previously described (Jin et al., 2019). Briefly, the collected supernatants were serially diluted from 10^–1^ to 10^–11^ with Opti-MEM medium that contained 2 μg/mL TPCK-treated trypsin. MDCK cells in 96-well plates were infected with diluted virus. Three days later, HA titer of each well was tested with 0.5% chicken red blood cells and TCID_50_ was calculated according to Reed-Muench assay.

### Neuraminidase Inhibition Assay

The inhibition of NA activity by 1G8 was measured with enzyme-linked lectin assay (ELLA) and 4-(methylumbelliferyl)-*N*-acetylneuraminic acid (Mu-NANA) assay as previously described (Wan et al., 2016).

In ELLA, mixtures of serial-diluted mAb and predetermined viruses were incubated in fetuin (Sigma-Aldrich, Shanghai, China) coated wells at 37°C for 16 h. After washing with PBST for six times, peanut agglutinin conjugated with peroxidase (PNA-HRP) (Sigma-Aldrich, Shanghai, China) were added and incubated at room temperature for 2 h. The plates were washed with PBST for six times, followed by addition of tetramethylbenzidine (TMB) substrate. The reaction was finally stopped with 1% SDS and absorbance at OD_650_ was read.

In Mu-NANA assay, mixtures of serial-diluted mAb and predetermined viruses were incubated in black 96-well plate at 37°C for 30 min. Mu-NANA substrate (Sigma-Aldrich, Shanghai, China) was added and incubated for 1 h at 37°C. The reaction was finally stopped with 0.2 M Na_2_CO_3_ and read with excitation range 350–365 nm and emission range 440–460 nm.

### Mice Experiments

For mice experiments, the BALB/c mice were purchased from Experimental Animal Center of Yangzhou University (Yangzhou, China). All animal experiments were done in accordance with the institutional animal care guidelines, and the protocol (number 06R015) was approved by the Animal Care Committee at Yangzhou University.

In the prophylactic experiment, eleven 6-week-old BALB/c mice per group were first intraperitoneally injected with 5 mg/kg mAb, and 2 h later, mice were anesthetized with 0.2 mL 1.25% avertin by intraperitoneal injection and infected with rgH1N2(JSH1) and rgH1N2(PUMCH06), respectively, at a dose of 10^7^ TCID_50_ by intranasal inoculation. On day 3 and 6 post-infection, three mice from each group were euthanized, and lungs were collected and the viral load in the lungs was titrated in MDCK cells by TCID_50_ assay. The lung tissues with representative pathological changes collected at the sixth day post-infection in prophylactic experiment were fixed with 4% paraformaldehyde for histopathological analysis. The other five infected mice in per group were monitored daily for body weight loss and any clinical signs. The mice with the body weight loss more than 25% were euthanized.

In the therapeutic experiment, five 6-week-old BALB/c mice per group were anesthetized and infected with rgH1N2(JSH1) and rgH1N2(PUMCH06) with a dose of 10^8^ TCID_50_ by intranasal inoculation. Mice of each group received 5, 2.5, 1, 0.5 mg/kg mAb 1G8 or 5 mg/kg mAb A7E6 by intraperitoneal injection at 48 h post-infection. The body weight of each group was monitored daily. The mice with the body weight loss more than 25% were euthanized.

### Selection of Monoclonal Antibody Escape Mutants

Escape mutants of rgH1N2(PUMCH06) and JS06 H3N2 canine influenza virus (CIV) were selected with mAb 1G8 as previously reported (Wang et al., 2021). Briefly, 50 μL allantoic fluid of each virus was incubated with 0.5 mL mAb at 37°C for 30 min and then inoculated into 9-day-old SPF embryonated chicken eggs. Allantoic fluid of the virus was collected on 5th day post-inoculation and NA gene of each virus was amplified with RT-PCR assays for sequencings. Mutant viruses were cloned by limiting dilution in 9-day-old embryonated chicken eggs and plaque assay, followed by further NA sequencing.

### Sequence Alignments and Phylogenetic Analysis

Twenty-two NA amino acid sequences (accession number: ABP49330.1, AJM70556.1, ALM05426.1, ANG55885.1, ALT67802.1, AQS25225.1, AEM75969.1, ACD88721.1, ADP07897.1, AHZ43615.1, AGG81752.1, AGG83215.1, ASV60611.1, AGG8 2970.1, AGG83292.1, CAC69608.1, AKF35396.1, AQS26225.1, AFC35440.1, AFC35430.1, AKC43905.1, and AGX84936.1) belong to 11 different NA subtypes were analyzed using multiple sequence alignment by MEGA X.^1^ The phylogenetic tree was constructed with MEGA X in neighbor-joining method and 1000 boot-strap replicates.

### Statistical Analysis

The data analysis was performed by GraphPad Prism v.5 (GraphPad Software Inc.). All results of viral growth, NI assays and mouse experiments were indicated as the mean ± SEM.

## Results

### Monoclonal Antibody 1G8 Inhibits Enzymatic Activity of H9N2 and H3N2 Neuraminidase

**FIGURE 1:**
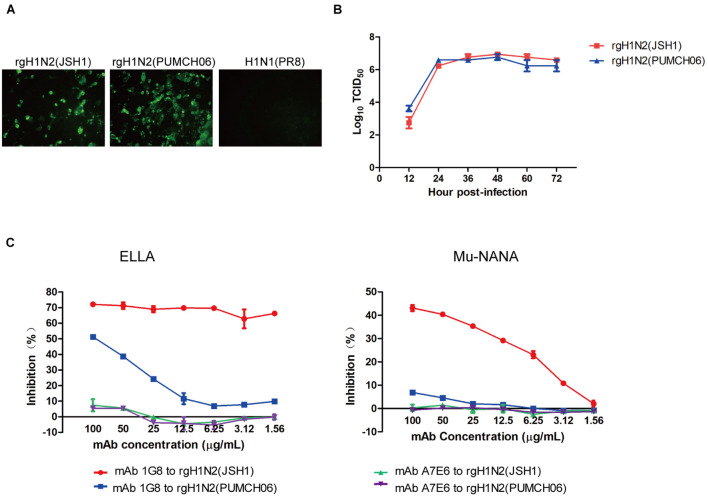
Generation of rgH1N2 viruses and reactivity to mAb 1G8. **(A)** Reactivity of mAb 1G8 to rgH1N2(JSH1), rgH1N2(PUMCH06), and H1N1(PR8) viruses in IFA. **(B)** Viral growth kinetics of rgH1N2 viruses in A549 cells. **(C)** NI activity of mAb 1G8 to rgH1N2 viruses measured by ELLA and Mu-NANA assay. The mAb A7E6 was used as negative control. All data of viral growth kinetics and NI assay were performed with Graphpad Prism v.5 and represented as mean ± SEM.

Rescued reassortant viruses rgH1N2(PUMCH06) and rgH1N2(JSH1) were generated with NA gene of JSH1 H9N2 virus or PUMCH06 H3N2 virus and 7 other genes of PR8 H1N1 virus. Rescued viruses were tested with mAb 1G8 in immunofluorescence assay (IFA) (Figure 1A). The IFA results showed that mAb 1G8 reacted with both rgH1N2(PUMCH06) virus and rgH1N2(JSH1) virus but not PR8 virus, indicating that mAb 1G8 could recognize NAs of not only H9N2 AIV but also H3N2 HSIV.

rgH1N2(PUMCH06) and rgH1N2(JSH1) exhibited similar replication kinetics in A549 cells (Figure 1B), which indicated that recombinant viruses with either avian-origin or human-origin N2-subtype NA can grow well in A549 cells. rgH1N2(JSH1) viruses had slightly lower reproduction level than rgH1N2(PUMCH06) in A549 cells at first 24 h postinfection, but rgH1N2(JSH1) viruses posed comparatively higher titers of viruses after 36 h postinfection.

In NI assays, mAb 1G8 showed significant inhibition effect on rgH1N2(JSH1) virus both in ELLA and Mu-NANA assay (Figure 1C). Although mAb 1G8 posed weaker inhibition activity to NA of rgH1N2(PUMCH06) virus compared with that to rgH1N2(JSH1) virus, significant inhibition to NA of rgH1N2(PUMCH06) virus can take place at high concentration of mAb 1G8, especially in ELLA. In the negative control group, mAb A7E6 cannot inhibit NA activity of the rgH1N2 viruses even at very high concentration. Results of NI assays implied that mAb 1G8 can inhibit NA activity of not only current H9N2 AIVs but also prevalent H3N2 HSIVs.

### Monoclonal Antibody 1G8 Protects Mice Against Viruses Bearing H9N2 and H3N2 Neuraminidase

**FIGURE 2:**
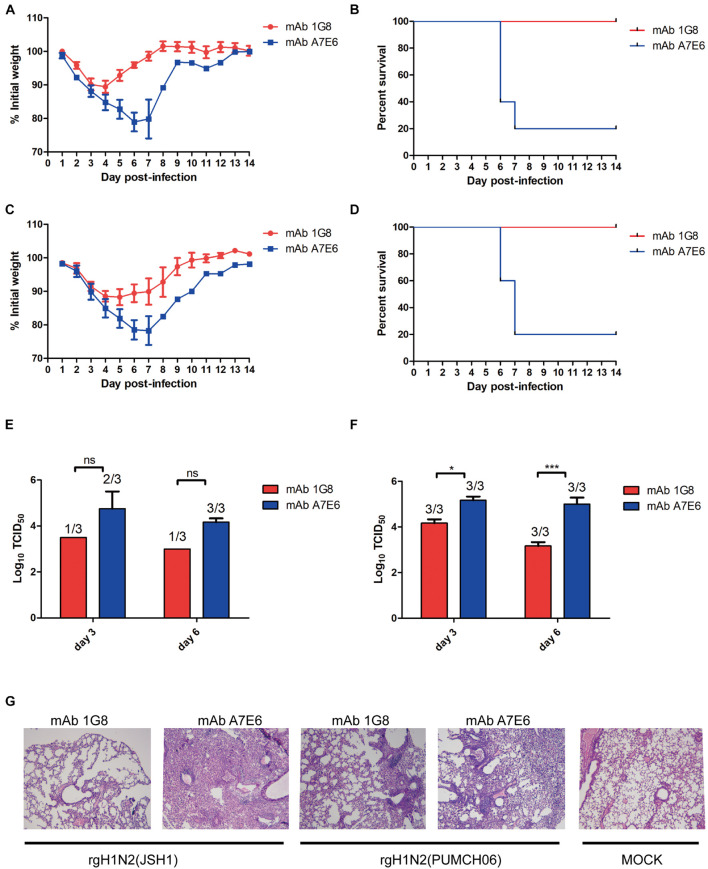
*In vivo* protective effect of mAb 1G8 in prophylactic experiment. The mean percentage of the mice body weight **(A)** and the survival curves **(B)** of BALB/c mice (*n* = 5 per group) treated with mAb 1G8 or mAb A7E6 after challenge with 10^7^ TCID_50_ rgH1N2(JSH1) viruses. The mean percentage of the mice body weight **(C)** and the survival curves **(D)** of BALB/c mice treated with mAb 1G8 or mAb A7E6 after challenge with 10^7^ TCID_50_ rgH1N2(PUMCH06) viruses. Viral titers in lungs of mice treated with mAb 1G8 or mAb A7E6 were determined on days 3 and 6 postinfection of 10^7^ TCID_50_ rgH1N2(JSH1) viruses **(E)** or rgH1N2(PUMCH06) viruses **(F)**. The *p*-values were calculated using two-way ANOVA using Dunnett’s multiple comparisons test with a 95% CI (ns, not significant; *p < 0.05; ***p < 0.0001). **(G)** Histological analysis of lungs from uninfected mice and infected mice treated with mAb 1G8 or mAb A7E6. The photos were taken in 100-fold magnification.

To test if mAb 1G8 has protective activity *in vivo*, we assessed the efficacy in prophylaxis and therapy of mAb 1G8 against recombinant H1N2 viruses in mice. In the prophylactic experiment, 1G8 provided 100% protection at a dose of 5 mg/kg for mice challenged with 10^7^ TCID_50_ of rgH1N2(JSH1) or rgH1N2(PUMCH06) (Figures 2B,D). The continuous weight losses of mice in 1G8 group infected with two viruses were only observed at the first 4 days, and the body weight recovered quickly right about less than 90% (Figures 2A,C). Whereas, the body weight of the negative control group, which was treated with mAb A7E6, continuously decreased on the first week and only one mouse in each group recovered on the second week.

The administration of mAb 1G8 also resulted in a reduction of viral load in lungs of the challenged mice (Figures 2E,F). Especially for the 1G8 group infected with rgH1N2(JSH1) virus, two of three mice were viral positive in lungs at third day postinfection, and only one viral positive in lungs collected at sixth day postinfection was detected. Consistent to viral load in lungs, the histopathological analysis results of infected mice showed that 1G8 resulted in less lesions and inflammations in lungs at sixth day postinfection compared with the control mAb (Figure 2G). The 1G8-treated mice had only mild alveolitis, while the negative control mAb A7E6-treated mice had severe pulmonary interstitial pneumonia and alveolitis. The alveolar structure of control mAb-treated mice is destroyed compared with the 1G8-treated mice, especially in those challenged with rgH1N2(JSH1).

**FIGURE 3:**
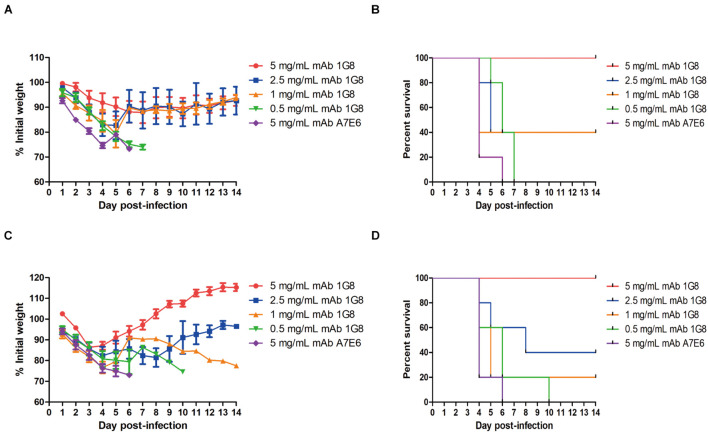
*In vivo* protective effect of mAb 1G8 in therapeutic experiment. **(A)** The mean percentage of mice body weight of BALB/c mice (*n* = 5 per group) challenged with 10^8^ TCID_50_ rgH1N2(JSH1) viruses and treated with mAb 1G8 (5, 2.5, 1, and 0.5 mg/kg) or mAb A7E6 (5 mg/kg). **(B)** The survival curves of BALB/c mice (*n* = 5 per group) challenged with 10^8^ TCID_50_ rgH1N2(JSH1) viruses and treated with mAb 1G8 (5, 2.5, 1, and 0.5 mg/kg) or mAb A7E6 (5 mg/kg). **(C)** The mean percentage of body weight of BALB/c mice challenged with 10^8^ TCID_50_ rgH1N2(PUMCH06) viruses and treated with mAb 1G8 (5, 2.5, 1, and 0.5 mg/kg) or mAb A7E6 (5 mg/kg). All data were performed with Graphpad Prism 5 and represented as mean ± SEM. **(D)** The survival curves of BALB/c mice challenged with 10^8^ TCID_50_ rgH1N2(PUMCH06) viruses and treated with mAb 1G8 (5, 2.5, 1, and 0.5 mg/kg) or mAb A7E6 (5 mg/kg).

In the therapeutic experiment, 1G8 still provided 100% protection at a dose of 5 mg/kg for mice challenged with 10^8^ TCID_50_ rgH1N2(JSH1) virus or rgH1N2(PUMCH06) virus (Figures 3B,D). Lower doses of mAb 1G8 did not provide 100% protection. At a dose of 2.5 mg/kg 1G8, only 40% of the animals survived. However, mice treated with lower doses of 1G8 showed slower weight loss and death in contrast with mice treated with the negative control mAb A7E6 (Figures 3A,C).

### Mutations at Amino Acid Position 199 of Neuraminidase Help Virus Escape From Monoclonal Antibody 1G8

To identify if 1G8 targets the same epitope in NA of H3N2 HSIV as previously reported for H9N2 AIV (Wan et al., 2016), escape mutant of rgH1N2(PUMCH06) selected by 1G8 was characterized. The K199R (N2 numbering) mutation in NA was found in selected escape mutant of rgH1N2(PUMCH06) virus.

**FIGURE 4:**
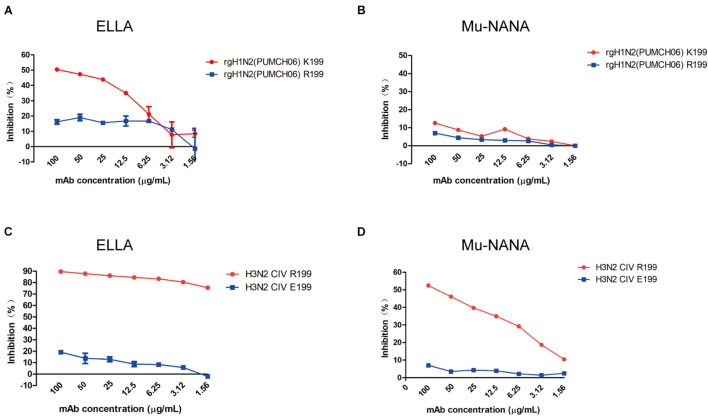
Neuraminidase inhibition ability of mAb 1G8 to mutant viruses. The NI activity of mAb 1G8 to rgH1N2(PUMCH06) viruses with K199 or R199 in NA were measured in both ELLA **(A)** and Mu-NANA assay **(B)**. The NI activity of mAb 1G8 to JS06 CIVs with R199 or E199 in NA were measured in both ELLA **(C)** and Mu-NANA assay **(D)**. All data of NI assay were performed with Graphpad Prism v.5 and represented as mean ± SEM.

The NI activity of 1G8 to the mutant virus was also measured with ELLA and MuNANA assays (Figures 4A,B). Compared with the rgH1N2(PUMCH06) virus containing K199 in NA, substituting R199 reduced the inhibitory effect of 1G8. Residue K199 is conserved in the current H3N2 HSIV, while R199 is a dominant residue in NA of H3N2 CIV. However, mAb 1G8 can still well react with JS06 H3N2 CIV, which has a R199 in NA. Therefore, another escape mutant with R199E mutation in NA of H3N2 CIV was selected with mAb 1G8. Whereas, mAb 1G8 showed very strong NI effect on WT H3N2 CIV but very weak NI effect on the selected mutant of the H3N2 CIV with an R199E mutation in NA in both ELLA and Mu-NANA assay (Figures 4C,D). All results indicated that, K199R mutation is crucial for H3N2 HSIV to escape from mAb 1G8. While E199 is the key residue for H3N2 CIV to escape from mAb 1G8, which is consistent with our previous report in H9N2 AIV (Wan et al., 2016).

### *N*-Linked Glycosylation at Residue 200 Does Not Shield Epitope(s) Containing Residue 199

**FIGURE 5:**
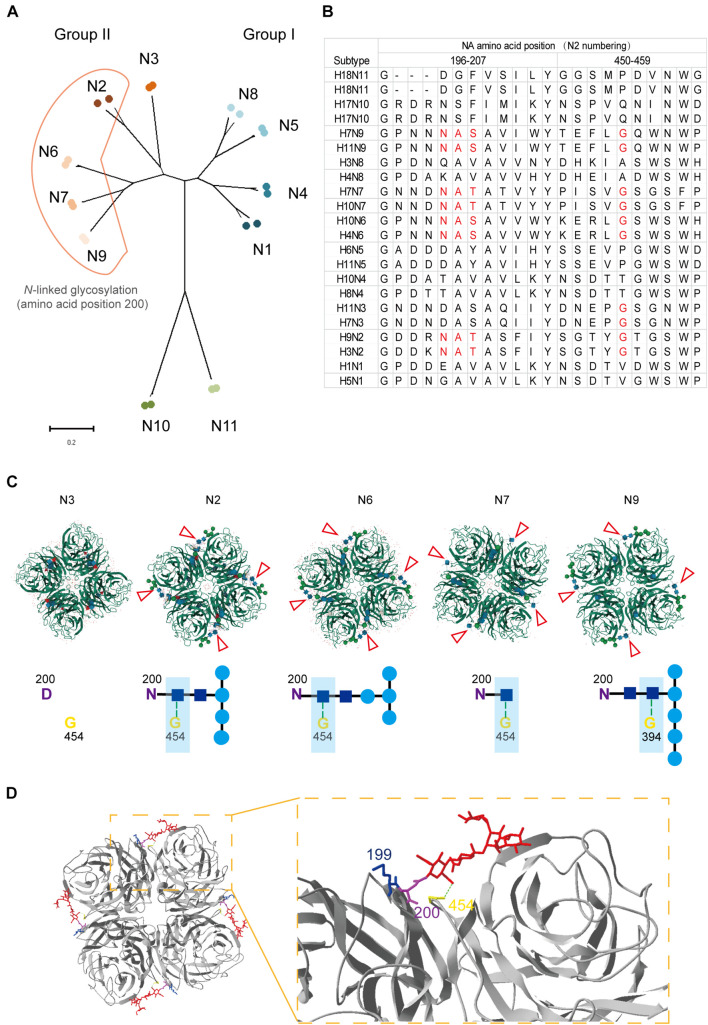
Structural and alignment analysis of residue 199 in NAs. **(A)** The distribution of *N*-linked glycosylation at amino acid position 200 in NA subtypes was shown in phylogenetic tree. **(B)** NA amino acid sequences of 22 strains belong to 11 subtypes was analyzed by alignment with MEGA X. The amino acids from position 196 to 207 and 450 to 459 were shown. Amino acids involved in forming the position 200 *N*-linked glycosylation site and residue 454 were marked in red. **(C)** Locations of different *N*-linked glycosylation modification at position 200 in group II NAs (PDB: 4HZV, 1NN2, 4QN6, 4QN3, and 3NN9) were analyzed online with MoI viewer in RCSB PDB. The glycosylation modification in NA tetramer was shown above and marked with red triangles. The hydrogen bond between *N*-acetylglucosamine (NAG) and glycine at position 454 or 394 was shown with green dot line below. **(D)** Status of residue 199 in case of *N*-linked glycosylation modification at residue 200 was analyzed by Swiss PDB Deep-viewer. The structure of N2 (PDB:4GZX) was used for analysis. The sugar chain was marked in red. Residues 199, 200, and 454 were individually labeled with blue, purple, and yellow. Hydrogen bond between NAG and G454 (or G394) is shown with green dotted line.

Position 199 locates close to the NA active center and plays an important role in inducing protective antibodies against H3N2 HSIVs (Gulati et al., 2002; Kirkpatrick Roubidoux et al., 2021). Human-derived antibodies targeting position 199 in NA showed broadly protective effect against multiple NA subtypes of viruses *in vivo* (Stadlbauer et al., 2019). However, the *N*-linked glycosylation sites at positions 200 to 202 were conserved in all group II NA subtypes except N3 (Figures 5A,B), which may form sugar chain modification and block the antibody binding with residue 199 by steric hindrance. Interestingly, structure analysis result shows that the sugar chain of each *N*-linked glycosylation at residue 200 in N2, N6, N7, and N9 is fixed on the adjacent NA monomer but not an active glycan shield. Although group II NA type virus except the N3 subtype virus have different glycosylation modifications at N200, the *N*-acetylglucosamine (NAG) of each sugar chain bind with the G454 or G394 in adjacent NA monomer by the hydrogen bond (Figure 5C). The alignment result also shows that G454 is highly conserved in viruses of all group II NA subtypes (Figure 5B).

Although, the gap among monomers and epitopes around residue 454 are buried by the sugar chain, which may result in less cross-reactive bodies against group I and group II NA subtype viruses. While the fixed sugar chain of the *N*-linked glycosylation at position 200 leaves enough space for the residue 199 being fully exposed to the antibodies (Figure 5D). All in all, residue 199 is able to take part in inducing protective antibodies without the influence by the *N*-linked glycosylation at residue 200.

## Discussion

N2-subtype influenza viruses have caused two pandemic disasters in humans. The first one was caused by H2N2 influenza virus in 1957, which had an avian-born NA (Kilbourne, 2006). H3N2 influenza virus resulted in the second one and evolved into seasonal flu since its first outbreak in 1968 (Kilbourne, 2006). H9N2 AIV cannot transmit from human to human, but H9N2 infections have been reported in humans (Peacock et al., 2019). Although both H9N2 AIV and H3N2 HSIV belong to the N2 subtype, there used to be few reports on the same antigenic epitopes or antigenic structures between two NAs.

We previously reported a mAb 1G8 against NA of H9N2 AIV, which possesses NI activity (Wan et al., 2016; Wang et al., 2020). We now further identified that mAb 1G8 cannot only cross-react with NAs of H9N2 AIV and H3N2 HSIV but also have NI activity to them in NI assays.

In our mice challenge model, mAb 1G8 also shows protective effect in both prophylactic and therapeutic experiments. Lower viral load and less lesions in mouse lungs were detected with treatment of 5 mg/kg 1G8. Less and slower progress of deaths occurred in mice treated with 1G8 compared with control groups in the therapeutic experiment. Mutations and *N*-linked glycosylation have been reported for NA of H9N2 AIVs after 2011 and H3N2 HSIVs circulating since 2016 and contributed to escape from humoral immunity (Wan et al., 2019; Powell and Pekosz, 2020; Wang et al., 2021), while mAb 1G8 cannot only show well reactivity and inhibition activity to the NAs of these viruses, it also poses great protection against rgH1N2 viruses challenge.

Our previous research identified mutations D198N and K199E in NA which can help H9N2 AIV escape from mAb 1G8 (Wan et al., 2016). In this study, mutation K199R was selected in NA of H3N2 HSIV with mAb 1G8, which indicates that position 199 is also a crucial binding site for mAb 1G8 reacting with human-origin N2-subtype NA. Both mAbs B10 and Mem 5 interact with residue 199 in NA of H3N2 HSIV by the CDR2 within the heavy chain (Venkatramani et al., 2006; Wan et al., 2019). Moreover, broadly protective human antibodies 1G04, 1E01, and 1G01 also bind residue 199 in NA by CDR2 in the light chain (Stadlbauer et al., 2019), which indicates epitopes with residue 199 are crucial for NA-specific protective antibodies.

Antibodies against NA of seasonal H1N1 viruses can provide sufficient protection *in vivo* against the lethal H5N1 AIV challenge (Sandbulte et al., 2007; Frobert et al., 2010; Rockman et al., 2013). Furthermore, influenza infection can induce broadly cross-reactive and protective NA antibodies (Chen et al., 2018). In conclusion, this mAb 1G8 can also inhibit NA activity and show protection in mice challenged with rgH1N2 viruses, which would be a good candidate for developing antibody drugs to both AIVs and HSIVs. Moreover, we find that residue 199 in NA is not buried by the *N*-linked glycans at position 200 and is fully exposed for the binding of antibodies. The epitope targeted by mAb 1G8, which includes position 199 can be further studied in the future for development of ideal universal influenza vaccine.

## Data Availability Statement

The datasets presented in this study can be found in online repositories. The names of the repository/repositories and accession number(s) can be found in the article/supplementary material.

## Ethics Statement

The animal study was reviewed and approved by the Animal Care Committee at Yangzhou University.

## Author Contributions

FW and ZW wrote the manuscript. FW, YW, JW, ZW, and HF performed the experiment and data analysis. FW, AQ, WG, ZW, JY, KQ, and HS designed the study. All authors have reviewed and approved the final vision of this manuscript.

## Conflict of Interest

The authors declare that the research was conducted in the absence of any commercial or financial relationships that could be construed as a potential conflict of interest.

## Publisher’s Note

All claims expressed in this article are solely those of the authors and do not necessarily represent those of their affiliated organizations, or those of the publisher, the editors and the reviewers. Any product that may be evaluated in this article, or claim that may be made by its manufacturer, is not guaranteed or endorsed by the publisher.

## Footnotes

1
www.megasoftware.net
